# Metabolic Syndrome in Iranian Youths: A Population-Based Study on Junior and High Schools Students in Rural and Urban Areas

**DOI:** 10.1155/2013/738485

**Published:** 2013-05-30

**Authors:** Alireza Ahmadi, Mojgan Gharipour, Fatemeh Nouri, Nizal Sarrafzadegan

**Affiliations:** ^1^Isfahan Cardiovascular Research Center, Isfahan Cardiovascular Research Institute, Isfahan University of Medical Sciences, Isfahan, Iran; ^2^Molecular and Cellular Biology, Isfahan Cardiovascular Research Center, Isfahan Cardiovascular Research Institute, Isfahan University of Medical Sciences, Isfahan, Iran; ^3^Cardiac Rehabilitation Research Center, Isfahan Cardiovascular Research Institute, Isfahan University of Medical Sciences, Isfahan, Iran

## Abstract

*Aim*. The present population-based study aimed to assess prevalence of metabolic syndrome and itsrelated components in Iranian youth in the different sex, age, and residential subgroups. *Method*. Overall, 1039 junior high school and 953 high school students were selected using multistage random sampling. Fasting blood sugar, total cholesterol, triglyceride, high-density lipoprotein cholesterol (HDL-C), and low-density lipoprotein cholesterol (LDL-C) levels were determined. Trained individuals measured waist circumference and blood pressure. Subjects with MetS were selected according to two definitions provided by the IDF and de Ferranti. *Results*. Among girls in intervention area, hypertriglyceridemia was more prevalent in rural than in urban areas using IDF definition. Significant differences were observed between boys in rural and urban areas regarding some components of metabolic syndrome including hypertriglyceridemia and high waist circumference. Besides, boys who are residents in urban areas had higher blood pressure, as well as higher waist circumference, than boys in rural areas. *Conclusion*. Our youth population is at significant risk of developing metabolic syndrome, and the pattern of this phenomenon seems to be discrepant in boys as well as in rural and urban areas probably due to the different lifestyle aspects, genetic factors, and racial differences.

## 1. Introduction

A renewed interest has been recently created in relation to metabolic syndrome (MetS) among children and adolescents because of its close relationship with childhood and adulthood obesity and other metabolic disturbances and cardiovascular diseases. Because of lack of timely diagnosing MetS in the youth, developing future atherosclerotic cardiovascular diseases and diabetes is expectable [1–4]. Although many attempts have been made to define MetS in adults, introducing strict and unique definition for this phenomenon in young population is now challenging leading to report different prevalence rates for this syndrome from various communities [5, 6]. According to the published information from the National Health and Nutrition Examination Survey (NHANES) done in the US, the prevalence rate of MetS in children varies widely depending upon ethnicity, weight, body mass index, age, and even sex [7]. The prevalence of MetS in the United States youths has been widely ranged based on the different ethnicities from 2.0% in African American adolescents to 5.6% in Hispanic adolescents [7]. In NHANES III and Bogalusa Heart Studies, MetS in youths was expressed as 4.2% in children and adolescents aged 12 to 19 years and 3.6% in youths aged 8 to 17 years, respectively [8]. In a recent systematic review including 109 papers, the overall prevalence of MetS in youths ranged 3%–10% using Adult-Treatment-Panel-(ATPIII-)based criteria and 1%–7% for International Diabetes Federation (IDF) definition criteria that was lowest and highest in European and Asian populations (3.3%–4.2%) and Middle Eastern and North American populations (4.2%–10%), respectively [9]. Furthermore, it seems that the prevalence of MetS may not be discrepant in both genders that were never higher for girls than boys in recent reports.

There as little information regarding prevalence as well as sex and age distribution state of MetS in Iranian youths. Some reports on Iranian youth population indicated prevalence of this syndrome in the range of 3.3% to 10.1% showing a high prevalence of the MetS in Iranian youths [10–13]. However, because of observed sexual, age, and ethnic disparities of the MetS and also due to the presence of ethnic diversity in our country, more studies on distribution of MetS in various rural and urban Iranian populations seem indispensable. The present population-based study aimed to assess prevalence of MetS and itsrelated components in Iranian youth in the different sex, age, and residential subgroups. 

## 2. Methods

The present community-based study was a large part of the Isfahan Healthy Heart Program (IHHP) conducted from 2000 to 2007 and was a community-based interventional program that was performed to prevent and control cardiovascular diseases and to promote healthy lifestyle. In this survey, the intervention community consisted of urban and rural regions in Isfahan and Najafabad, while Arak was studied as a control area. Arak, a county located 375 km north-west of Isfahan, was selected as a control area because it resembled the intervention areas in its socioeconomic, demographic, and health profiles and offered good cooperation file and offered good cooperation. Arak was monitored for evaluation purposes but did not receive any intervention [14, 15]. Adolescences was defined based on school grading as junior high school: 6th through 8th grade (ages 11–14) and high school: 9th through 12th grade (ages 14–18). 

### 2.1. Heart Health Promotion from Childhood (HHPC) Project

 HHPC project was performed as one of interventional projects of a comprehensive community-based program IHHP with school-based approach to improve lifestyle behavior and cardiometabolic risk factors among students in middle and high schools [16]. Briefly, HHPC public education was done through mass media, pamphlets, booklets, face-to-face meetings, proposing role models among students, arranging different competitions with the subject of healthy heart, serving healthy snacks, establishing healthy heart buffets, reinforcing healthy eating habits in schools, and gathering parents at least yearly to train healthy nutrition [16]. 

### 2.2. Participants

In the 3st phase, the situation was evaluated on 524 students in junior high schools (244 girls (212 urban, 32 rural), 280 boys (247 urban, 33 rural)) and 448 students in high school (223 girls (196 urban, 27 rural), 225 boys (202 urban, 23 rural)) selected from intervention area. In references area 515 students in junior high schools (256 girls (172 urban, 84 rural), 259 boys (166 urban, 93 rural)) and 505 students in high school (255 girls (171 urban, 84 rural), and 250 boys (166 urban, 84 rural)). School-based approach and multistage random-cluster sampling methods were conducted to choose 56 junior and high school students of different urban and rural areas based on population distribution in urban/rural ratio of intervention and reference area which was 70/30 and 60/40, respectively.

Complete information regarding sampling process has been presented elsewhere [16]. IHHP was done in three phases; characteristics of population was assessed during the first phase, and throughout the 2nd phase of the study, different interventions were performed in intervention area only on the basis of the results of the first study phase, whereas Arak remained as the reference area. In both intervention and reference areas, health-related lifestyle behaviors were determined through annual questionnaire-based surveys on independent samples, whereas physical examination and blood sampling were conducted in the first and final phases of the study. 

Healthy nutrition was one of the main fields of interventions in the IHHP program which was performed based on educational, environmental, and legislative strategies. Briefly, HHPC public education was done through mass media, pamphlets, booklets, face-to-face meetings, proposing role models among students, arranging different competitions with the subject of healthy heart, serving healthy snacks, establishing healthy heart buffets, reinforcing healthy eating habits in schools, and gathering parents at least yearly to train healthy nutrition. In this study the data of the last phase (after intervention) was used. 

The study protocol was approved by the Ethics Committee of the Isfahan Cardiovascular Research Center (a WHO collaborating center), and signed written informed consent was obtained from all subjects' parents. 

### 2.3. Anthropometric Measurements

 Anthropometric parameters including weight, height, and waist circumference were measured using standard tools. Height and weight were measured with subjects wearing light clothing and without shoes. Height was recorded to the nearest 0.1 cm. Weight was also measured to the nearest 0.1 kg using a balance-beam scale. The waist circumference was measured to the nearest 0.1 cm at the midpoint between the bottom of the rib cage and the top of the iliac crest at the end of exhalation. The participants' blood pressure was measured twice after a 5 min rest using the right hand, and the mean was recorded as their blood pressure.

### 2.4. Laboratory Measurements

 Blood lipids were measured enzymatically with the commercially available reagents (Cholesterol/HP, cat. no. 816302, and Triglycerides/GPO, cat. no. 816370, both from the Boehringer Mannheim). HDL cholesterol was measured in the clear supernatant after precipitating the other lipoproteins with heparin and MnCl_2_ (1.3 g/L and 0.046 mol/L, resp.) and removing excess Mn^2+^ by precipitation with NaHCO_3_. Fasting glucose was measured using the Glucose Standard Assay (Sigma Chemical, St. Louis) [14, 15].

### 2.5. MetS Definitions

 MetS in studied youths was defined using the two IDF and de Ferranti definitions. According to the new IDF definition [17], for a person to be defined as having the MetS, they must have at least three components of the following criteria: (1) waist circumference (WC) ≥ 90th percentile, (2) serum triglyceride ≥ 150 mg/dL, (3) HDL < 40 mg/dL, (4) systolic and diastolic blood pressure ≥ 90th percentile, and (5) fasting blood sugar (FBS) > 100 mg/dL.

MetS was also defined, using the criteria proposed by de Ferranti et al. [18], as three or more of the following variables and cutoff points: (1) WC ≥ 75th percentile, (2) serum triglyceride > 100 mg, (3) HDL < 40 mg/dL, (4) blood pressure ≥ 90th percentile, and (5) FBS > 100 mg/dL.

### 2.6. Statistical Analysis

Comparisons of the prevalence of MetS and its components based on grade, sex, intervention and reference in the urban and rural were done using chi-square and Fisher's exact test when more than 20% of cells with expected count of less than 5 were observed. Data were entered into EPI 2000 statistical software package (SPSS Inc., Chicago, IL). A 2-tailed *P* value less than 0.05 was considered statistically significant. *P* < 0.05 was considered significant.

## 3. Results

Sex distribution of different components of MetS according to two definition approaches in high schools of urban and rural areas is displayed in Table 1. In intervention group and among girls, except for hypertriglyceridemia that was more prevalent in rural than in urban areas using IDF definition, no significant differences were revealed in terms of the prevalence of other MetS components between the two regions. However, contrarily, significant differences were observed between boys in rural and urban areas regarding some components of MetS including hypertriglyceridemia and high waist circumference. As shown in Table 1, increased waist circumference was more observed in boys resident in urban than boys in rural regions; however, this discrepancy was not reported between girls in the two regions. Sex distribution of MetS in boys and girls of the junior high schools in both urban and rural regions had different patterns (Table 2). Girls in urban areas had higher blood pressure, had lower HDL level, and had higher waist circumference according to IDF definition compared with girls in rural areas. Besides, boys who are residents in urban areas had higher blood pressure, as well as higher waist circumference, than boys in rural areas. 

Examination of the proportion of youths meeting the IDF diagnostic criteria for MetS showed overall prevalence of 2.8% in girls and 6.6% in boys, while these prevalence rates based on de Ferranti definition in girls and boys were 10.9% and 14.4%, respectively. With respect to the prevalence of MetS in urban and rural areas, using IDF criteria led to the overall prevalence of 5.2% in urban youths and prevalence of 3.3% in rural youths, whereas employing de Ferranti definition definitive criteria obtained prevalence of 13.7% in urban areas and 9.0% in rural areas. 

## 4. Discussion

The major problem to diagnose MetS in both childhood and adulthood periods is unavailability of an accepted global definition of this phenomenon as well as different cutoff values for each component so that applying different definitive criteria including Cook [19], de Ferranti [20], Goodman [21], Weiss [22], Cruz [23], Ford [24], and IDF [25] has led to reporting various range of the prevalence rates from different societies. In this survey, we adopted IDF definition to determine the prevalence and distribution of metabolic syndrome among Iranian middle and high school students based on its convenience in epidemiological research and clinical practice, using de Ferranti's definition as a supplement. The results of this study suggest that regardless of weight categories of subjects, there is a high prevalence of this syndrome in both mentioned student groups compared to the published ranges from other populations. Also, a higher prevalence of different components of the MetS, including central obesity, hypertension, high triglycerides, low HDL-C, and high glucose, was revealed in our samples compared with other observations. In addition, our results demonstrated higher prevalence of central obesity and hypertension in boys resident in urban than boys in rural regions, which can be attributed to the lifestyles of adolescents at this age, for example, immobility [26], interest in processed food, and entertainment devices such as computers and television. On the other hand, high school students pay more attention to their appearance, shape, and weight. Beside, girls in urban areas had higher blood pressure, had lower HDL level, and had higher waist circumference according to IDF definition compared with girls in rural areas [10]. Many studies discussed the effect of urban-rural residency on CVD risk factors. 

The level of parent's education and family income may predict dietary changes during lifestyle modification program in developing countries with social and economic transitions [27]. Wang et al. recommended that higher income family, urban residency, and higher educated mothers are more likely to track poor eating habits. Although these mothers had better access to the media and higher knowledge about healthy eating, their behaviors demonstrated that they had no attention about higher fat foods [27]. 

In fact, Iranian youths tend to have a cluster of multiple components of the MetS that may be one of the most alarming public health issues facing our country. By applying as de Ferranti's criteria, the prevalence of metabolic syndrome in our middle and high school students was estimated 13.2% and 12.3%, considerably higher than that reported among in the western countries and Chinese population (about 10.0% and 6.6%, resp.) [28–32]. Meanwhile, the overall prevalence of MetS defined by IDF criteria was lower than that defined by de Ferranti's criteria. Only 4.2% to 5.3% of our participants were identified with MetS using the IDF definition while at least 2 to 3 times higher using de Ferranti's definition due to under estimating prevalence of MetS by IDF criteria compared with de Ferranti's definition [33]. However, employing two definitions led to estimating high prevalence of MetS in our children and adolescents probably due to inappropriate lifestyle features including unhealthy dietary habits, low physical activity, and even genetic factors. In our observation, the patterns of the prevalence of MetS and its-related components varied in boys and girls as well as in rural and urban areas probably due to the different lifestyle feature differences and genetic diversities. 

In order to understand the pathophysiology of MetS in children and adolescents, the focus of recent studies has been narrowed to insulin resistance and obesity [34]. In fact, main feature of MetS is attributed to insulin resistance, and weight loss seems to play an indirect role by increasing insulin sensitivity. On the other hand, high insulin levels over a long period of time results in increased rate of obesity, hyperlipidemia, and hypertension [35, 36]. In this regard, association between elements of MetS and excess adiposity has been shown even in children younger than 5 years [37, 38]. Decrease of insulin sensitivity can be appeared as a result of the detrimental effect of inflammatory molecules on the insulin signaling pathways leading to break down of the response to insulin [33]. Meanwhile, the changes in the pattern of hormonal secretion around puberty can also affect body fat, blood pressure, and lipid profile, predisposing children to this syndrome [33]. 

In summary, our youth population at significant risk of developing MetS and the pattern of this phenomenon seems to be discrepant in boys as well as in rural and urban areas probably due to the different in lifestyle aspects, genetic factors, and racial differences. 

## Figures and Tables

**Table 1:** Prevalence of different components of metabolic syndrome in high school students based on urbanization and intervention, reference area.

High school	Girls			Boys		
	Urban	Rural	*P* value	Urban	Rural	*P* value
Number of participants						
Intervention	196	27		202	23	
Reference	171	84		166	84	
High BP						
Intervention	19 (9.8)	0	0.14	32 (15.9)	1 (4.3)	0.21
Reference	50 (29.4)	14 (17.9)	0.055	35 (21.1)	19 (22.9)	0.74
High TG IDF						
Intervention	12 (6.2)	5 (18.5)	0.04	26 (13.0)	0	0.08
Reference	7 (4.8)	5 (6.0)	0.76	11 (6.6)	20 (23.8)	<0.001
High TG de Ferranti						
Intervention	74 (37.9)	14 (51.9)	0.17	78 (39.0)	3 (13.6)	0.019
Reference	52 (35.9)	39 (46.4)	0.11	57 (34.3)	61 (72.6)	<0.001
Low HDL						
Intervention	46 (23.6)	9 (36.0)	0.18	61 (30.5)	6 (30.0)	0.96
Reference	31 (21.4)	16 (19.0)	0.67	49 (29.5)	31 (36.9)	0.24
High FBS						
Intervention	10 (5.1)	3 (11.1)	0.198	26 (13.0)	1 (4.5)	0.49
Reference	1 (0.7)	1 (1.2)	1.00	1 (0.6)	0	1.0
High WC de Ferranti						
Intervention	40 (20.6)	8 (29.6)	0.29	64 (32.2)	0	0.001
Reference	29 (17.1)	15 (18.3)	0.81	41 (25.0)	3 (3.6)	<0.001
High WC IDF						
Intervention	18 (9.3)	4 (14.8)	0.32	33 (16.6)	0	0.03
Reference	10 (5.9)	3 (3.7)	0.55	16 (9.8)	0	0.003
Metabolic syndrome (IDF)						
Intervention	7 (3.6)	0 (0)	0.6	20 (10.1)	0 (0)	0.23
Reference	3 (2.1)	1 (1.2)	1.0	4 (2.4)	4 (4.8)	0.45
Metabolic syndrome (de Ferranti)						
Intervention	22 (11.3)	3 (11.1)	1.0	38 (19.1)	0 (0)	0.017
Reference	19 (13.0)	8 (9.5)	0.43	14 (8.4)	9 (10.7)	0.56

Data are expressed as number (%).

*P* value obtained from Chi-square test or if required.

**Table 2:** Prevalence of different components of metabolic syndrome in junior high school students urbanization and intervention, reference.

Junior high school	Girls			Boys		
	Urban	Rural	*P* value	Urban	Rural	*P* value
Number of participants						
Intervention	212	32		247	33	
Reference	172	84		166	93	
High BP						
Intervention	34 (16.2)	1 (3.1)	0.06	63 (25.7)	0 (0)	0.001
Reference	63 (36.6)	19 (24.1)	0.048	82 (49.4)	22 (23.9)	0.000
High TG IDF						
Intervention	29 (13.8)	3 (9.7)	0.78	19 (7.8)	4 (12.1)	0.496
Reference	30 (17.4)	6 (16.2)	0.86	27 (16.3)	13 (18.3)	0.70
High TG de Ferranti						
Intervention	99 (47.1)	15 (48.4)	0.89	99 (40.4)	15 (45.5)	0.58
Reference	103 (59.9)	27 (73.0)	0.14	72 (43.4)	46 (64.8)	0.003
Low HDL						
Intervention	67 (31.9)	4 (12.9)	0.030	62 (25.4)	11 (33.3)	0.33
Reference	29 (16.9)	14 (37.8)	0.004	43 (25.9)	17 (23.3)	0.67
High FBS						
Intervention	7 (3.3)	2 (6.5)	0.33	32 (13.1)	3 (9.1)	0.78
Reference	2 (1.2)	0 (0)	1.00	2 (1.2)	1 (1.4)	1.00
High WC de Ferranti						
Intervention	37 (17.6)	9 (29.0)	0.13	63 (25.7)	3 (9.4)	0.04
Reference	22 (12.9)	11 (13.1)	0.97	67 (40.4)	8 (8.6)	<0.001
High WC IDF						
Intervention	20 (9.5)	1 (3.2)	0.49	35 (14.3)	2 (6.3)	0.28
Reference	9 (5.3)	0 (0)	0.032	25 (15.1)	3 (3.2)	0.003
Metabolic syndrome (IDF)						
Intervention	7 (3.3)	1 (3.2)	1.00	15 (6.1)	2 (6.3)	1.00
Reference	3 (1.7)	3 (8.1)	0.07	18 (10.9)	2 (2.7)	0.036
Metabolic syndrome (de Ferranti)						
Intervention	19 (9.1)	2 (6.5)	1.00	36 (14.8)	3 (9.4)	0.59
Reference	19 (11.0)	6 (16.2)	0.40	38 (22.9)	4 (5.5)	0.001

Data are expressed as number (%).

*P* value obtained from chi-square test (or Fisher's exact test if required).
